# Research on temperature prediction model for key components in highspeed rail electrical cabinets based on LSTM

**DOI:** 10.1371/journal.pone.0355411

**Published:** 2026-08-17

**Authors:** Lihui Zhou, Xiansheng Liu, Shuai Jin

**Affiliations:** CRRC TANGSHAN Co., Ltd., Tangshan, China; Beijing Institute of Technology, CHINA

## Abstract

The temperature status of key components in high-speed rail electrical cabinets directly impacts the safety and reliability of train operations. To achieve accurate temperature prediction and early warning, this paper proposes a temperature prediction model based on Long Short-Term Memory (LSTM) networks. Addressing the current research gap and the limitations of traditional models in capturing long-term temporal dependencies in temperature data, a multivariate dataset was first constructed by collecting time-series temperature data of key components along with relevant environmental and operational parameters. Subsequently, comparative analyses were conducted by establishing RBF neural network, ARIMA, Prophet, and LSTM prediction models, systematically investigating the impact of LSTM network architecture parameters and time window settings on prediction accuracy. Experimental results demonstrate that the LSTM model significantly outperforms the comparison models in both prediction accuracy and stability, achieving superior performance in key metrics such as RMSE and MAE. The model effectively captures long-term trends and short-term fluctuations in temperature variations, providing reliable technical support for condition monitoring and fault warning of key components.

## 1. Introxsduction

With the rapid development of China's high-speed rail, the requirements for the safety and reliability of train operations are continuously increasing [1–5]. As a core component of the train's power system, the temperature status of key components inside the electrical cabinets of high-speed rail (such as relays) is an important indicator reflecting the health level of the equipment [6–8]. An abnormal temperature rise of components usually indicates potential hazards such as overload, poor contact, insufficient heat dissipation, or insulation aging. Failure to timely detect and remedy these issues may lead to system malfunctions or fires, severely compromising train operational safety. Therefore, achieving real-time and accurate prediction of the temperature of key components in electrical cabinets is of great significance for preventing failures, improving operation and maintenance efficiency, and ensuring the safe and efficient operation of high-speed rail [9,10].

In the field of temperature prediction, various methods have been explored. Traditional statistical approaches, such as the Autoregressive Integrated Moving Average (ARIMA) model, are interpretable and effective for stationary time series but struggle with nonlinear, non-stationary data driven by external operating conditions. Machine learning methods, including Radial Basis Function (RBF) neural networks, can approximate nonlinear mappings but are prone to overfitting when temporal dependencies are strong. More recently, deep learning models, particularly Long Short-Term Memory (LSTM) networks, have demonstrated superior capability in capturing long-term dependencies in sequential data. For example, Wang Yuanfei et al. [11] applied RBF to bearing temperature prediction, Yan Youjun [12] used multiple nonlinear regression for transformer temperature, and Song Jiayin [13] employed ARIMA for high-speed train bearing temperature. LSTM-based approaches have been successfully applied to transformer winding temperature prediction [14], substation equipment temperature monitoring [15], and motor temperature forecasting [16]. Furthermore, LSTM has been successfully applied to other time-series prediction tasks in related domains, such as remote condition monitoring of rail tracks using distributed acoustic sensing (DAS) with a deep CNN-LSTM-SW model, and feature-selection-based irradiance forecasting for stand-alone photovoltaic systems. These studies further demonstrate the strong capability of LSTM in capturing complex temporal dependencies under dynamic operational conditions [17–22].

Although LSTM has been applied to temperature prediction for transformers and substation equipment, to the best of our knowledge, no study has specifically addressed the temperature prediction of key components (e.g., relays) inside high-speed rail electrical cabinets, where strong coupling exists among temperature, operating speed, cumulative mileage, and ambient conditions. This paper aims to fill this gap by proposing a dedicated LSTM-based prediction framework that explicitly models these multivariate interactions.

To verify the model's effectiveness, Radial Basis Function (RBF) neural networks, autoregressive Integrated Moving Average (ARIMA) models, and Prophet models are constructed as benchmark models. Based on actually collected operational data, each model is trained, validated, and tested, and their prediction performances are comprehensively compared and analyzed from multiple evaluation metrics. Furthermore, this paper delves into the impact of key parameters of the LSTM network (e.g., hidden layer size, learning rate, time step) and time series window parameters (input window length, prediction step) on prediction accuracy, providing references for model optimization and engineering applications. Experimental results show that, compared with the benchmark models, the LSTM model proposed in this paper performs better in terms of both prediction accuracy and anti-interference capability.

## 2. The construction of the temperature prediction model

### 2.1 The principle of the temperature prediction model

The temperature prediction model for key components in the electrical cabinets of high-speed rail, proposed in this paper, is based on Long Short-Term Memory (LSTM) networks. The model takes historical temperature sequences as the core input and integrates external operational parameters closely related to temperature variations, jointly constructing a time-series prediction framework capable of characterizing the temperature-operation condition coupling relationship. During the rolling prediction process, external parameters are synchronously updated based on availability and physical characteristics: cumulative operating mileage is directly calculated by an odometer or time integration and is a deterministic quantity; operating speed is based on the predetermined planned speed curve of the train as future input; ambient temperature (inside the vehicle) is treated as a constant within the short-term prediction window.

In terms of specific implementation, the Long Short-Term Memory (LSTM) network first performs feature extraction and temporal modeling on historical temperature data and synchronized external parameters, thereby generating the temperature prediction value of key components at the next moment. Subsequently, this prediction result is treated as a new known quantity, which, together with the updated external parameters, is fed into the model to continue the rolling prediction for the next moment. By leveraging this “prediction-update-reprediction” iterative mechanism, the model can continuously deduce the temperature evolution trajectory over multiple future moments and maintain the stability and coherence of prediction results over longer time scales. Compared with traditional static methods, this approach better reflects the dynamic response of temperature to operating conditions and its long-term dependency characteristics, providing more reliable data support and decision-making basis for early identification of abnormal temperature rises, implementation of fault early warning, and optimization of operation and maintenance scheduling.

**Fig 1:**
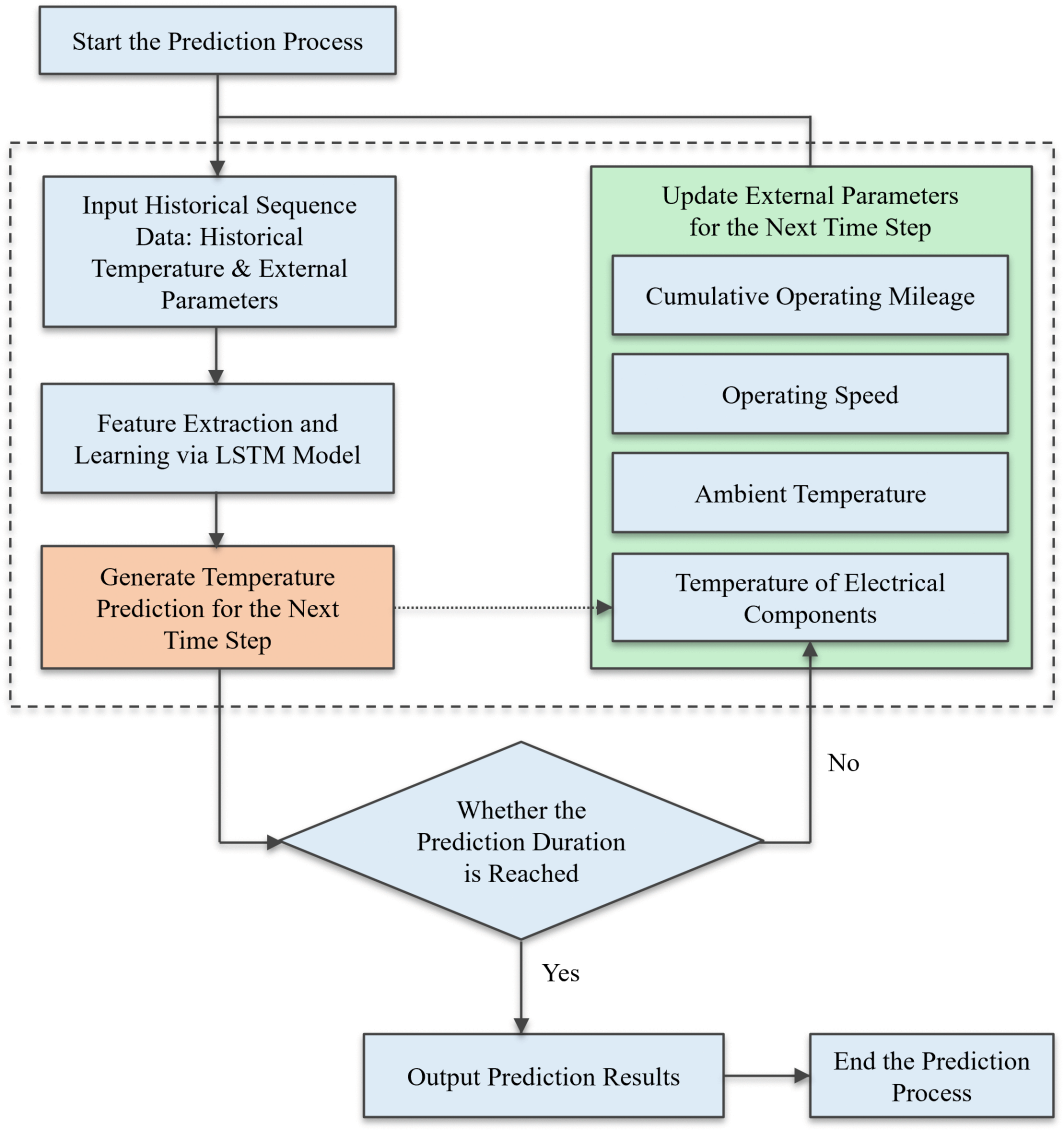
Temperature prediction process diagram for electrical components.

It is acknowledged that the use of a predetermined planned speed curve as the future input for operating speed *vt* simplifies real-world uncertainties such as traffic adjustments or weather-induced deceleration. However, within the short-term prediction window (e.g., 5 minutes), speed variations are typically limited and follow predictable patterns (e.g., approaching a station). For longer horizons or higher uncertainty scenarios, the model can be extended by integrating real-time speed predictions or robust interval forecasts, which is left for future work. Fig 1 illustrates the temperature prediction process diagram for electrical components.

### 2.2 LSTM model structure

The Recurrent Neural Network (RNN) is capable of capturing temporal dependencies when processing sequence data, but it suffers from a severe long-term dependency problem. Specifically, as the number of time steps increases, the network struggles to effectively transmit and retain early-stage information, leading to gradient vanishing or gradient explosion. The Long Short-Term Memory (LSTM) model adopted in this study is an improved architecture of RNNs. By introducing a gating mechanism, it can effectively control the retention and forgetting of information, thereby better modeling long-term dependencies in long sequences. It is widely applied in fields such as time series prediction and natural language processing.

**Fig 2:**
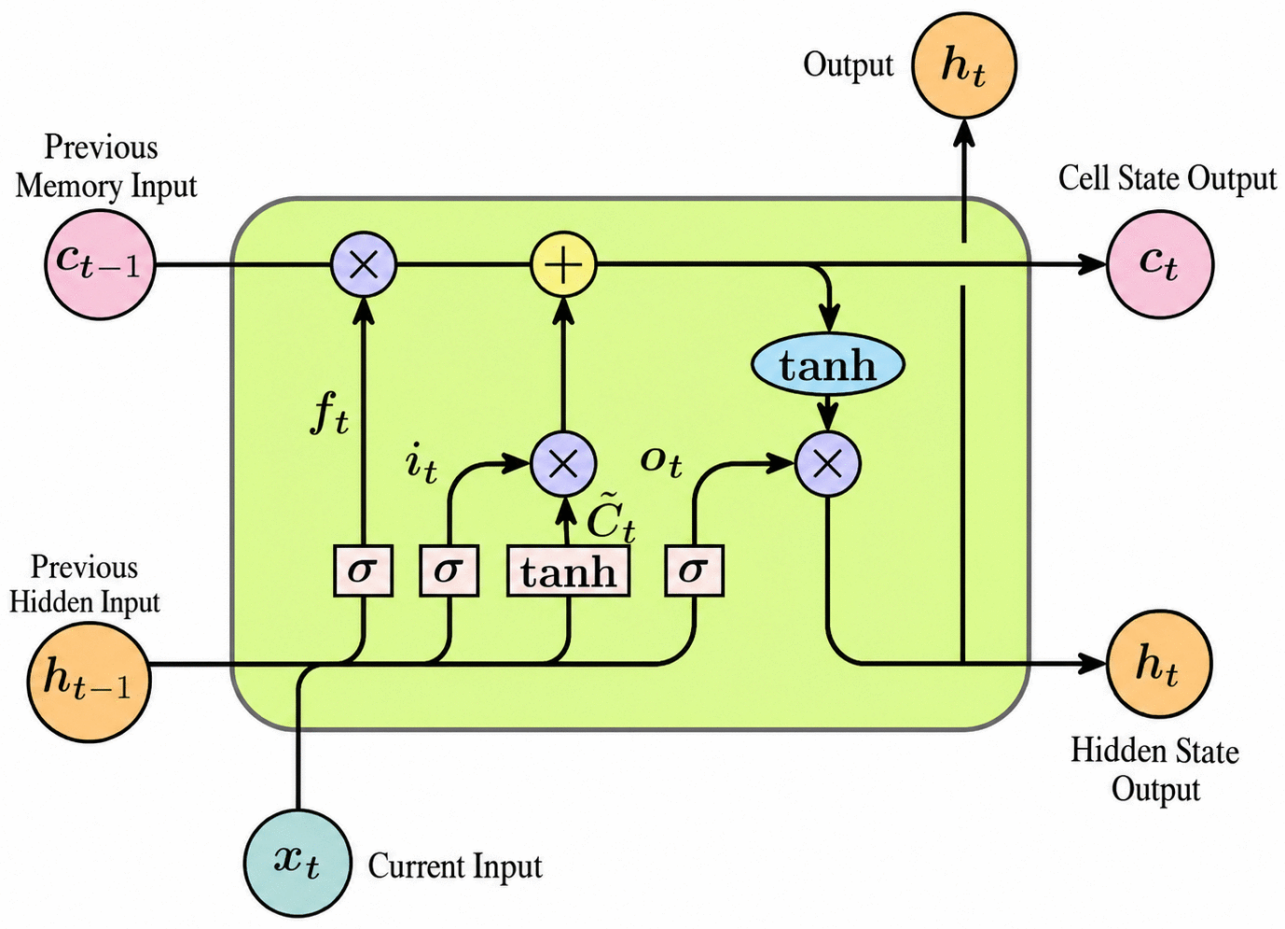
Structure of LSTM.

LSTM employs three key gating mechanisms (as illustrated in Fig 2) — specifically, the forget gate (which regulates which information in the prior-time-step cell state *Ct* − 1 ought to be discarded), the input gate (which decides which new information from the current input *xt* should be written into the cell state), and the output gate (which controls how the current hidden state *ht* is generated from the cell state and determines the information to be externally output). By means of these gating mechanisms, LSTM selectively determines what information should be retained, updated, or output within the cell state, thus realizing effective modeling of long-sequence information.

In the context of temperature prediction for electrical components, the forget gate *ft* determines how much past temperature information should be retained, which is crucial for capturing long-term trends such as gradual warming during sustained operation. The input gate *it* controls the influence of recent temperature changes and external parameters (e.g., a sudden speed increase), enabling rapid response to short-term fluctuations. The output gate *ot* regulates the final prediction based on the updated cell state. This gating mechanism inherently addresses the long-term dependency problem that plagues conventional RNNs.

(1) The relationship between the forget gate and the input gate is formalized in Equation (1):


$$\begin{array}{c} f_{t}=\sigma (W_{f}\;\cdot \;[h_{t-\text{1}},x_{t}]+b_{f})\text{\textasciitilde{}}i_{t}=\sigma [W_{i}\;\cdot \;[h_{t-\text{1}},x_{t}]+b_{i\;})\text{\textasciitilde{}}\tilde{C_{t}}=tanh[W_{c}\cdot \;[h_{t-\text{1}},x_{t}]+b_{c}) \end{array}$$ (1)

In the formula: *ft* denotes the forget gate, which controls the proportion of memory from the previous time step that is retained; *it* represents the input gate, governing the proportion of new information written; $\tilde{C_{t}}$ is the candidate memory value, indicating potential new information; *σ* is the sigmoid activation function, outputting weights within the range of 0 ~ 1; tanh is employed to generate the candidate state, with an approximate range of −1 ~ 1.

(2) The relationship between the update of the memory cell and the output of the hidden state is formalized in Equation (2):


$$\begin{array}{c} C_{t}=f_{t}\cdot \;{∁}_{t-\text{1}}+\;i_{t}\cdot \;\tilde{C_{t}}\;h_{t}=o_{t}\cdot \;tanh(C_{t}) \end{array}$$ (2)

In the formula: *C_t_* is the memory cell state at the current time step (representing long-term memory); *ht* is the hidden state at the current time step (output to the subsequent layer); *o_t_* is the output gate (regulating the content of the hidden state's output).

### 2.3 Model evaluation

The temperature prediction problem belongs to the regression computation problem. To evaluate the error magnitude of predicted values, this paper selects core metrics including the coefficient of determination (*R*²), root mean square error (RMSE), mean absolute error (MAE), mean absolute percentage error (MAPE), and maximum absolute relative error (MaxRE), with the aim of comprehensively measuring the prediction accuracy and fitting ability of the model.

The coefficient of determination evaluates the goodness of fit between the model's predicted values and actual values. The closer it is to 1, the higher the goodness of fit of the approximate model, with the expression given by Equation (3). The root mean square error (RMSE) is defined as the square root of the mean of the squared differences between predicted values and actual values. A smaller RMSE indicates higher credibility of the approximate model, with the expression given by Equation (4). The mean absolute error (MAE) is given by Equation (5). The mean absolute percentage error (MAPE) is defined as the average absolute percentage difference between predicted and actual values, as given in Equation (6). Additionally, the maximum absolute relative error (MaxRE) is used to evaluate the local prediction capability, as given in Equation (7).


$$\begin{array}{c} R^{\text{2}}=\frac{\sum_{i=\text{1}}^{n_{t}}{\left({\hat{y}}_{i}-\overset{―}{y}\right)}^{\text{2}}}{\sum_{i=\text{1}}^{n_{t}}{\left(y_{i}-\overset{―}{y}\right)}^{\text{2}}} \end{array}$$ (3)

$$\begin{array}{c} \text{RMSE}=\sqrt{\frac{\text{1}}{n_{t}}\sum_{i=\text{1}}^{n_{t}}{\left({\hat{y}}_{i}-y_{i}\right)}^{\text{2}}} \end{array}$$ (4)

$$\begin{array}{c} \text{MAE}=\frac{\text{1}}{{\text{n}}_{\text{t}}}\sum_{\text{i}=\text{1}}^{{\text{n}}_{\text{t}}}\left|{\hat{y}}_{i}-y_{i}\right| \end{array}$$ (5)

$$\begin{array}{c} \text{MAPE}=\frac{\text{1}}{{\text{n}}_{\text{t}}}\sum_{\text{i}=\text{1}}^{{\text{n}}_{\text{t}}}\left|\frac{{\hat{y}}_{i}-y_{i}}{y_{i}}\right| \end{array}$$ (6)

$$\begin{array}{c} \text{MaxRE}=\text{max}\left(\frac{\left|{\hat{y}}_{i}-y_{i}\right|}{y_{i}}\right)\times \text{100}\% \end{array}$$ (7)

where *nt* is the number of sample points for testing the accuracy of the approximate model; ${\hat{y}}_{i}$ is the predicted value of the i-th sample point; $\;y_{i}$ is the actual value of the i-th sample point; $\overset{―}{y}$ is the mean of the actual values of all sample points.

### 2.4 Temperature prediction model implementation

The specific implementation workflow of the temperature prediction model is as follows:

Data Processing: Construct the input vector *xt* (as shown in Table 1) based on the main influencing factors of the temperature of key components, which mainly includes historical temperature sequences and external operating parameters.

**Table 1:** Input vector.

Serial Number	Variable	Variable Content	Remarks
1	*T*_*t*-*k* + 1:*t*_	Electrical Component Temperature	A total of historical temperature data
2	*S*_*t*_	Cumulative Operating Mileage	Calculated from the odometer or time integration
2	*v*_*t*_	Operating Speed	Derived from the established planned speed curve
4	*T*_0_	Ambient Temperature	Treated as a constant within the short – term prediction window

(1) For each variable, compute the mean *μ* and variance $\sigma^{\text{2}}$, and save these statistics. Subsequently, normalize each variable using the formula for z-score standardization

$$\begin{array}{c} x^{norm}=\frac{x-\mu }{\sigma } \end{array}$$ (8)

To derive multiple short – sequence samples$\;\left\{X_{i},y_{i}\right\}\;$from the long – term time series, we first segment the long – term time series based on the time step (determined by the sampling window length k and the step size h ). In this case,$\;X_{i}\in \;R^{k\times d}$ (where d represents the dimension of variables), and$\;y_{i}\;$is either the single – step label$\;T_{t+\text{1}}\;$or the multi – step label$\;T_{t+\text{1}:t+h}\;$.After that, the training set, validation set, and test set are divided in chronological order to avoid information leakage: the training set is used for model fitting, the validation set is used for hyperparameter tuning or early stopping, and the test set is used for the final evaluation. In order to improve the generalization ability, while not disturbing the internal temporal order of the short sequences, we randomize the arrangement order of the short – sequence samples. This can reduce the temporal correlation among different short sequences and prevent overfitting.

(2) Network Construction: An LSTM-based time series prediction network is constructed using PyTorch, composed of stacked LSTM layers and a Linear output layer. The LSTM layers are designed to extract temporal dependencies and long-term memory from historical temperature data and external parameters. The regression output layer maps the hidden state at the last time step to either the temperature at the next time step (single-step prediction) or an h-step temperature vector (multi-step prediction). To account for the “temperature - process coupling”, at each time step, $[T_{t},S_{t},v_{t},T_{\text{0}}]\;$are jointly input. For multi – step rolling prediction, single – step results can be output with recursion carried out during the inference phase; for sequence – to – sequence tasks, multi – step results within a fixed – length window can be directly output.
(3) Model Training: The model is deployed on a GPU. Training data are input to the LSTM in batches for forward and backward propagation, where the loss function is chosen as MSELoss (Mean Squared Error Loss). The optimizer adopts Adam, combined with learning rate decay. Regularization includes L2 weight decay and LSTM dropout. To mitigate error accumulation during multi-step rolling prediction, we employ a scheduled sampling strategy during training: with a probability that decays over training epochs, the predicted temperature at the previous time step is used instead of the ground truth as input for the next step. This forces the model to learn to correct its own errors. The early stopping strategy halts training when the validation loss shows no significant decrease over consecutive rounds; subsequently, the network parameters are reverted to those at the point of minimal validation loss and saved.
(4) Implementation of Rolling Prediction

During the inference stage, an iterative process of “prediction – update – reprediction” is adopted:

1) Obtain the standardized input sequence$\;X_{t-k+\text{1}:t}\;$(including temperature and external parameters) over the most recent k time steps;
2) Forwardly derive the standardized temperature at the next time step, denoted as$\;\overset{norm}{\underset{t+\text{1}}{\hat{T}}}\;$;
3) Conduct inverse normalization to acquire$\;{\hat{T}}_{t+\text{1}}=\overset{norm}{\underset{t+\text{1}}{\hat{T}}}\cdot \sigma_{T}+\mu_{T}\;$;
4) Concatenate$\;{\hat{T}}_{t+\text{1}}\;$(treated as a “known quantity”) into the input queue, and synchronously update external parameters following predefined rules:$\;s_{t+\text{1}}=s_{t}+\Delta_{s}$; $v_{t+\text{1}}\;$is sourced from the scheduled speed profile;$\;T_{\text{0}}\;$remains unchanged within the short – term window;
5) Iteratively execute steps 1–4 to obtain the future temperature predictions$\;\left\{{\hat{T}}_{t+\text{1}},\cdots ,{\hat{T}}_{t+n}\right\}\;$.

This recursive mechanism guarantees dynamic responsiveness to variations in operational conditions and the continuous transmission of long-term dependencies. For inference, multi-step predictions are generated recursively, but the number of recursive steps is limited (e.g., ≤ 6 steps for 5-minute ahead warning) to control error propagation. Empirical validation shows that the accumulated RMSE grows sublinearly with prediction steps due to the model's strong short-term correlation capture.

(5) Model Evaluation: The test set (the last segment of data retained chronologically) is fed into the trained model to obtain standardized predictions, which are then reconstructed using$\;\sigma_{T}\;$and$\;\mu_{T}\;$saved in Step(1):

$$\begin{array}{c} Y=Y_{norm}\cdot \sigma_{T}+\mu_{T} \end{array}$$ (9)

Subsequently,$\;R^{\text{2}}$,RMSE, MAPE and MaxRE are calculated across sub – time intervals (acceleration/uniform motion/deceleration) and working conditions (different ambient temperatures). This evaluates the model's robustness and error accumulation characteristics under diverse scenarios.

## 3. Validation of the LSTM-Based temperature prediction model using real-world vehicle data

### 3.1 Real vehicle operation temperature data acquisition

**Fig 3:**
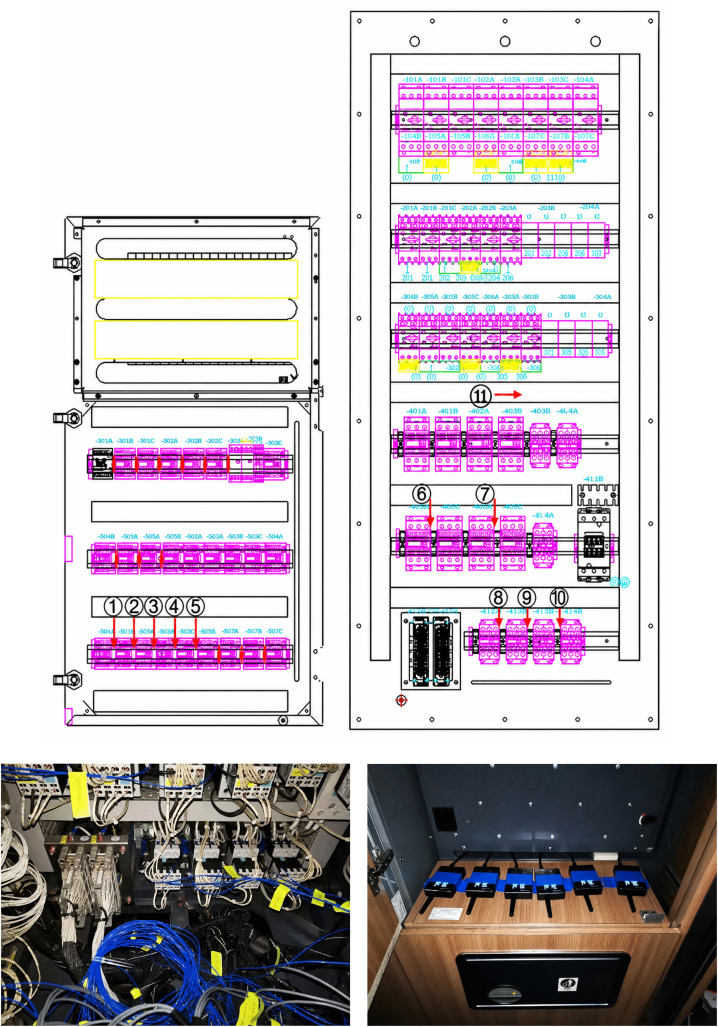
Deployment of temperature monitoring system for highspeed rail electrical cabinets.

To validate the effectiveness of the electrical component temperature prediction model, this study conducted a real vehicle temperature acquisition experiment. The test subject was the electrical cabinet in Car No. 02 of a Fuxing CR400BF Electric Multiple Unit (EMU) operating on the main line. The test equipment utilized a SmartMeter temperature detection recorder, in conjunction with external patch-type temperature probes (model PT1000, accuracy ±0.3°C, resolution 0.1°C), to achieve multi-point synchronous acquisition, with data transmitted in real-time via a 4G network. The patch-type probes were fixed to the surface of the electrical components using high-temperature polyimide tape, ensuring close contact between the probe and the measured point during the test. The ambient temperature inside the vehicle was simultaneously recorded by an onboard sensor with an accuracy of ±0.5°C. The layout of the measurement points and the on-site environment are shown in Fig 3, with a total of 10 temperature acquisition points in this test.

During the on-site test, the sensor deployment and recorder installation were first completed, followed by setting the sampling frequency to 1 time/min and enabling the automatic transmission function. Throughout the test, the electrical cabinet was maintained in normal operation, with temperature data continuously acquired for approximately 12 hours. The external ambient temperature during the experiment ranged from 18°C to 26°C, while the inside-vehicle ambient temperature varied between 22°C and 30°C. The vehicle's cumulative operating mileage, real-time operating speed, and ambient temperature were obtained after being transmitted back to the maintenance platform via the high-speed train's onboard system.

It should be noted that although the sampling frequency was set to 1 sample per minute and the total acquisition duration was approximately 12 hours, the final dataset contains 726 samples. This slight deviation arises because the recorder remained active for an additional 6 minutes before departure and after parking, capturing transient thermal responses during vehicle startup and shutdown phases. These extra samples are retained to enrich the representation of boundary conditions.

**Fig 4:**
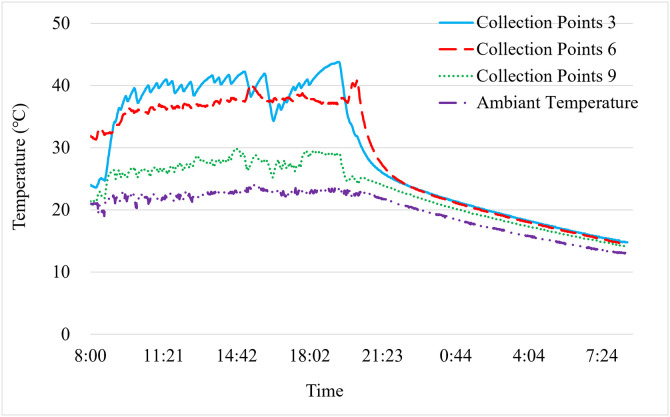
Time series diagram of collection points and environmental temperature. (a) the vehicle’s cumulative operating mileage. (b) the real – time operating speed.

**Fig 5:**
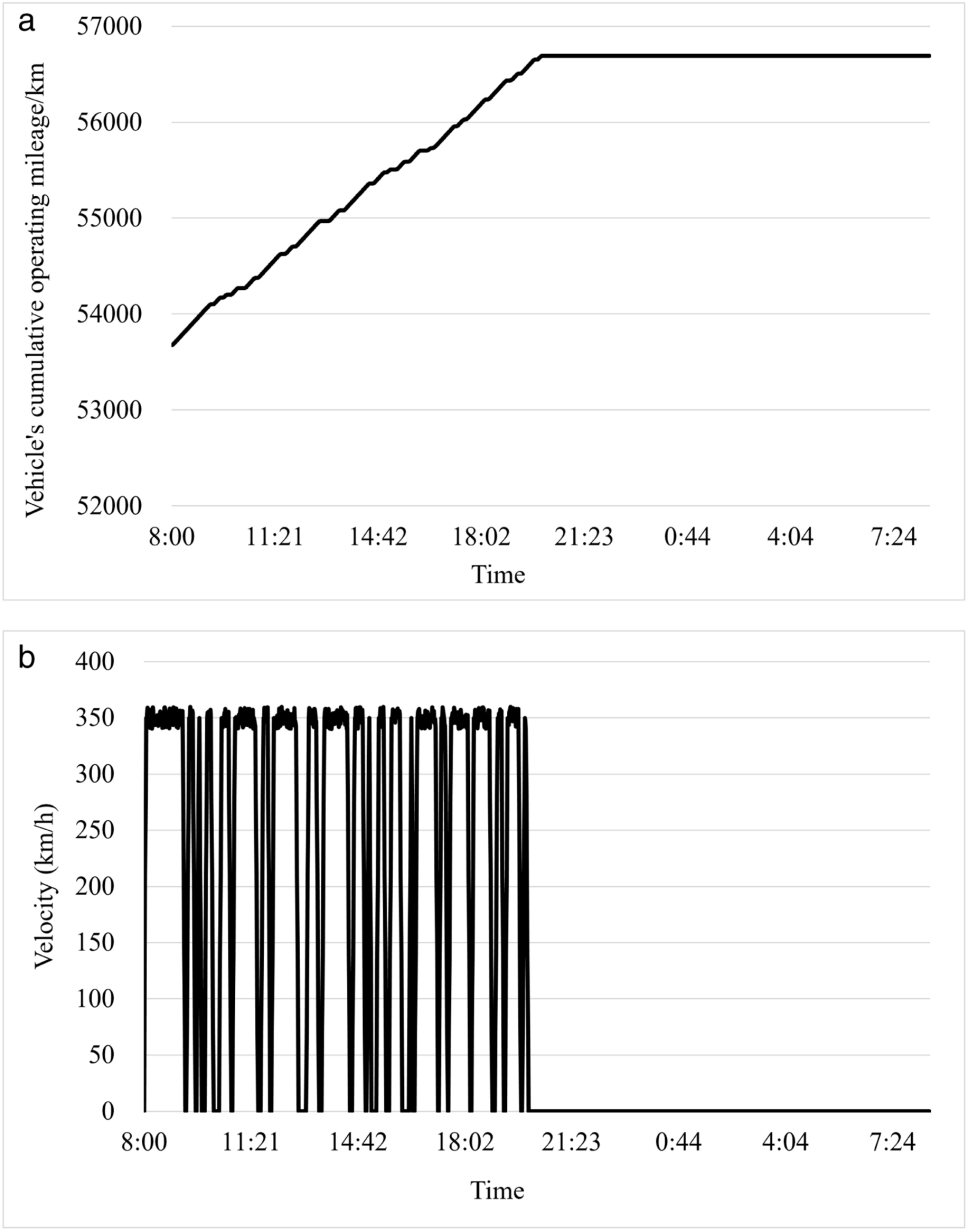
Accumulated mileage and real-time operating speed of vehicles.

Partial typical temperature curves, e.g., the time-series curves of temperatures at Collection Points 3, 6, and 9 and the ambient temperature curve, are shown in Fig 4. The vehicle's cumulative operating mileage and real-time operating speed are illustrated in Fig 5.

As can be observed from Fig 4, throughout the vehicle's entire operational cycle, the temperature at each collection point demonstrates dynamic variation patterns that are closely associated with the vehicle's operational states. During the vehicle's initial start-up stage, the temperature at each collection point increases as the electrical components function. In the sustained operation stage, the temperature at each collection point stays at a relatively elevated level and fluctuates in response to the vehicle's start-stop actions (including station entry, arrival, and departure). When the vehicle halts operation, the electrical components stop generating heat, and the heat gradually disperses, leading to the temperature returning to the ambient temperature.

Combining the cumulative operating mileage and real-time operating speed curves shown in Fig 5, it is evident that temperature variations exhibit a high degree of synchronization with operating speed: during periods of higher operating speeds, temperatures are generally elevated with intensified fluctuations; when the speed decreases, temperatures responsively exhibit a corresponding decline. This synchronized variation pattern further indicates that the thermal state of the components is directly modulated by train operating conditions, and thus the temperature prediction model must effectively capture this dynamic coupling relationship.

In summary, Figs 4 and 5 comprehensively illustrate the entire evolution process of the temperature of key electrical components in the electrical cabinet with the vehicle's operational states. Notably, the data from the operation stage serve as effective samples rich in dynamic features for model training, while the observation of temperature decline after vehicle halt provides important evidence for validating the causal relationship between temperature and operating conditions. Subsequent modeling will focus on the data from the operation stage to enable accurate temperature prediction under online conditions.

### 3.2 Validation of the LSTM-Based temperature prediction model

To validate the effectiveness of the LSTM model in temperature prediction for key electrical components in the electrical cabinet of high-speed trains, this section takes Collection Point 3 as an example and focuses on using data from the vehicle operation stage for model training and validation. The reason for selecting Collection Point 3 as the research object is that, as can be seen from Fig 4, this measurement point exhibits the most significant temperature fluctuations during operation. It can more fully reflect the dynamic variations of component temperatures with operating conditions, thereby imposing higher requirements on the sensitivity and robustness of the prediction model.

Based on the temperature data from the operation stage experimentally acquired and synchronously recorded parameters such as cumulative operating mileage, real-time operating speed, and ambient temperature, this study constructs a multivariate dataset for time-series prediction. The dataset has a sampling interval of 1 minute, and each sample contains historical temperature sequences along with corresponding external operating parameters. The dataset comprises 726 samples, equivalent to approximately 12 hours of experimental data (including startup and shutdown transients).

The dataset is partitioned into a training set, a validation set, and a test set at proportions of 70%, 15%, and 15%, respectively. The training set is used for model parameter learning, the validation set for hyperparameter tuning and convergence performance monitoring, and the test set serves as independent data for final performance evaluation. This partitioning strategy simultaneously ensures effective model training and validation of its generalization capability.

The model is trained by adopting the mean squared error (MSE) loss function and the Adam optimization algorithm. The network is configured with the following parameters: 2 stacked LSTM layers; hidden layer sizes of 64 (first layer) and 32 (second layer); dropout probability of 0.1; time step of 14; maximum number of iterations set to 100; and an early stopping mechanism that limits additional iterations to 15.

**Fig 6:**
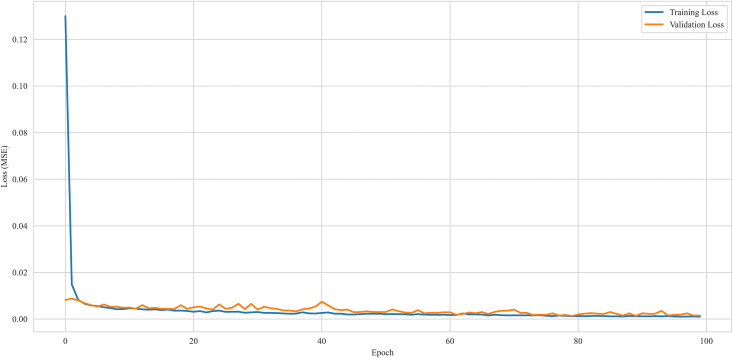
Model loss rate curve.

As can be observed from Fig 6, the model training loss declines sharply. After 10 iterations, the loss value stabilizes approximately. Training terminates after 30 iterations, with the final training loss reaching 0.25 and the validation loss being 0.05.

In the model evaluation stage, the primary evaluation metrics employed are the Root Mean Square Error (RMSE), Mean Absolute Error (MAE), and Coefficient of Determination (*R*²) to comprehensively assess the performance of the LSTM model in the temperature prediction task for key electrical components in the electrical cabinet of high-speed trains (see Table 2). Experimental results demonstrate that: the model's prediction performance on the test set reveals strong capability in capturing temperature variation trends, with a small overall deviation between predicted and true values and particularly high fitting accuracy near the mean value; it also indicates the model's good interpretability. Furthermore, on the validation set, the model maintains strong generalization capability on unseen validation data not involved in training, thereby validating the effectiveness of the LSTM model in capturing temporal features and long-term dependencies of temperature.

**Table 2:** LSTM model evaluation results.

Dataset	MAE	RMSE	*R*²
Training Set	0.71	0.87	0.95
Validation Set	1.26	1.44	0.81

**Fig 7:**
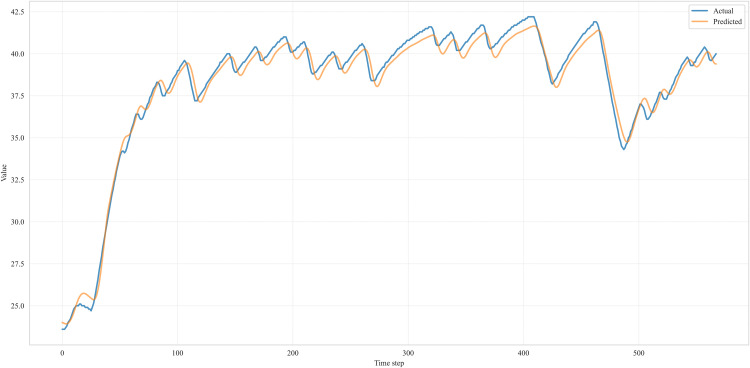
Training set temporal comparison curve.

**Fig 8:**
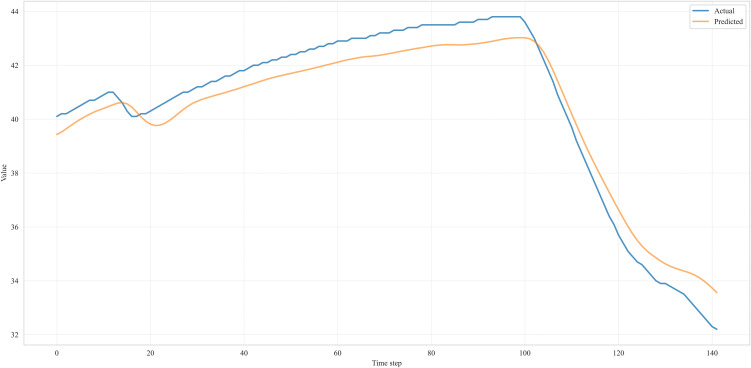
Validation set time-series comparison curve.

To further visually demonstrate the model's fitting performance and actual prediction capability, this study plotted time-series comparison curves of actual temperature values versus predicted values on the training set and validation set (see Figs 7 and 8). As can be observed from the figures, the model can effectively track the variation trends of actual temperatures on both the training set and validation set, with the predicted curves exhibiting a high overall degree of agreement with the true values. Particularly during periods with relatively stable temperature variations, the two nearly overlap, indicating that the model achieves high prediction accuracy for temperature dynamics under conventional operating conditions. At some moments of temperature fluctuations or abrupt changes, although the predicted values show slight deviations, the overall trends remain consistent, reflecting the model's effective modeling capability for temporal dependency relationships.

**Fig 9:**
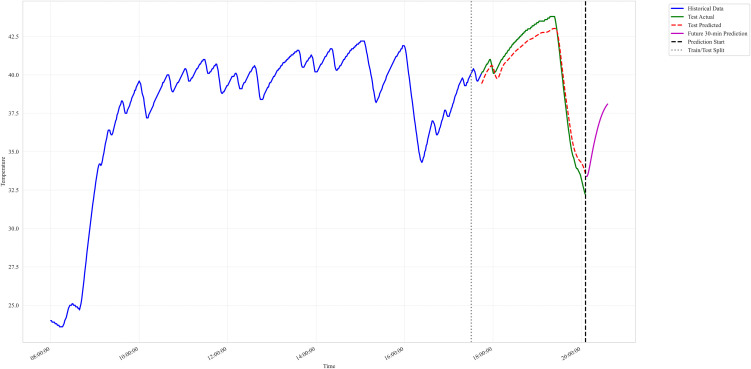
LSTM complete time series prediction results.

Additionally, this study also presents a complete time-series prediction graph (see Fig 9) that integrates historical observation data, actual and predicted values of the test set, and 30-minute future prediction results, aiming to comprehensively evaluate the model's practical application value and prospective prediction performance. The figure clearly distinguishes historical data, test actual values, test predicted values, future predicted values, and the prediction starting point using different colors or line styles. Among these, the test predicted values maintain high temporal consistency with the actual values during the test period, further validating the model's generalization capability on known data. More importantly, based on the integrated information from historical and test data, the model can generate reasonable predictions for the temperature variation trend over the next 30 minutes. The overall trend of the prediction curve is stable and consistent with historical variation patterns, and the selection of the prediction starting point is appropriate. This indicates that the model not only possesses favorable short-term prediction accuracy but also has certain medium-to-long-term trend inference capabilities, providing a scientific basis for thermal state warning and operation and maintenance decisions of electrical equipment. Integrating the quantitative evaluation metrics and the above visualization results, the constructed LSTM model demonstrates favorable fitting capability, generalization performance, and practical application potential in the temperature prediction task for key electrical components in the electrical cabinet of high-speed trains. It can provide reliable technical support for real-time monitoring and intelligent diagnosis of equipment operating states.

### 3.3 Multi-scenario and multi-point validation

To further evaluate the robustness of the proposed LSTM model, we extended the validation to two additional measurement points (Points 6 and 9) under three typical operating conditions: acceleration (speed increasing > 1 km/h per minute), constant-speed (speed variation < 5 km/h), and deceleration (speed decreasing > 1 km/h per minute). Table 3 summarizes the RMSE, *R*², and MAPE for each condition.

**Table 3:** Prediction performance under different operating conditions and measurement points.

Measurement Point	Condition	RMSE (°C)	*R*²	MAPE (%)
Point 3	Acceleration	1.52	0.79	2.34
Point 3	Constant-speed	0.87	0.94	1.12
Point 3	Deceleration	1.23	0.85	1.89
Point 6	Constant-speed	0.92	0.92	1.24
Point 9	Constant-speed	0.79	0.96	0.98

The results show that the model performs best under constant-speed conditions, where temperature variations are smooth and strongly correlated with recent history. During acceleration and deceleration, the RMSE increases moderately, indicating that the model captures most but not all of the transient thermal response. Performance across different points remains consistent, confirming that the model generalizes well to multiple components inside the same electrical cabinet.

## 4. Model comparison and parameter optimization analysis

### 4.1 Comparative analysis of different prediction models

To comprehensively validate the effectiveness and superiority of the LSTM model in the temperature prediction task for key electrical components in the electrical cabinet of high-speed trains, this study selected three typical prediction models for comparative experiments: the classical machine learning model Radial Basis Function (RBF) neural network, the decomposable time-series model Prophet with Bayesian inference (which is not a deep learning model), and the traditional statistical model Autoregressive Integrated Moving Average (ARIMA). Each model was trained and tested under identical dataset partitioning and training conditions, and the comparative results are shown in Table 4.

**Table 4:** Evaluation results table of different models.

Model	Dataset	RMSE	*R*²
LSTM	Validation Set	0.87	0.95
	Test Set	1.44	0.81
RBF	Validation Set	3.2	0.99
	Test Set	1.335	−0.48
Prophet	Validation Set	0.375	0.926
	Test Set	11.84	−115.26
ARIMA	Validation Set	0.154	0.987
	Test Set	1.03	0.12

The RBF neural network exhibits excellent performance on the training set (RMSE = 3.2, *R*² = 0.99), while its performance deteriorates sharply on the test set, with *R*² dropping to −0.48. This typical overfitting behavior arises because RBF models lack explicit temporal modeling and treat each sample independently. The temperature series of electrical components is highly non-stationary, with dynamics strongly modulated by operating speed and cumulative mileage. RBF's localized Gaussian kernels cannot generalize to unseen operating condition distributions, leading to dramatic performance collapse when the test set contains different speed patterns.

The Prophet model demonstrates good fitting capability on the training set (RMSE = 0.375, *R*² = 0.926), but its prediction performance degrades severely on the test set, where RMSE increases to 11.84 and *R*² drops to −115.26. Prophet assumes that time series can be decomposed into trend, seasonality, and holiday components with additive or multiplicative interactions. However, the temperature of electrical components in high-speed rail cabinets is not driven by calendar seasonality but by real-time operational conditions (speed, mileage, ambient temperature). When the test set exhibits a different speed profile, Prophet incorrectly attributes temperature variations to its predefined seasonal components, resulting in extreme errors.

As a representative of traditional time series methods, the ARIMA model shows strong linear fitting capability on the training set (RMSE = 0.154, *R*² = 0.987), but its generalization performance is notably insufficient on the test set (RMSE = 1.03, *R*² = 0.12). ARIMA captures short-term autocorrelation effectively in the training set but generalizes poorly to the test set because it relies on a linear autoregressive structure, whereas the actual temperature dynamics are nonlinear, especially during acceleration and deceleration phases where temperature responds nonlinearly to speed changes. The linear assumption fails to extrapolate beyond the training data distribution.

In contrast, the LSTM model exhibits the most balanced performance on both the training set and test set: with RMSE = 0.87 and *R*² = 0.95 on the training set, and RMSE = 1.44 and *R*² = 0.81 on the test set, indicating good generalization capability without overfitting. Compared with the other three models, the LSTM achieves the highest *R*² on the test set, with a significant performance gap relative to the others.

Based on the above comparative analysis, when processing temperature data of key electrical components in the electrical cabinet of high-speed trains—characterized by complex temporal dependencies and nonlinear features—the LSTM model exhibits stronger robustness and prediction accuracy. Its unique gating mechanism can effectively capture the long-term dependencies of temperature variations with operating conditions while avoiding overfitting, thereby holding significant advantages in practical applications. This result validates the applicability and advanced nature of the LSTM model in the temperature prediction task for key electrical components in the electrical cabinet of high-speed trains.

### 4.2 LSTM model parameter optimization analysis

This section, based on the same dataset as in Section 4.1, further conducts an optimization study on the internal parameters of the LSTM model—including network structure parameters (number of hidden layer units, Dropout rate) and time window parameters (input window length, prediction step size)—aiming to determine the optimal configuration suitable for temperature prediction for key electrical components in the electrical cabinet of high-speed trains.

#### 4.2.1 Impact of network structure parameters on prediction accuracy.

To systematically evaluate the individual impact of each architectural parameter, we designed an ablation study by varying one parameter at a time while keeping others fixed. The prediction performance of the LSTM model is closely related to its network structure. To explore the impact of different structural configurations on temperature prediction accuracy, this study selected seven representative LSTM network structures for comparative experiments, with specific configurations shown in Table 5. Each model was trained and tested under identical dataset partitioning (80% for training and 20% for testing), and the prediction performance metrics on the test set are presented in Table 6.

**Table 5:** LSTM network structure parameters.

Number	Model description	Network structure (Number of layer units)	Dropout rate
1	Benchmark	[64,32]	0.1
2	Large	[128,64]	0.1
3	Small	[32,16]	0.1
4	Medium	[64,64]	0.1
5	High Dropout	[64,32]	0.2
6	Low Dropout	[64,32]	0.1
7	Large	[128,32]	0.1

**Table 6:** Prediction results of different network structures (test set).

Number	*R*²	MSE	RMSE	MAE
1	0.84	1.73	1.32	1.11
2	0.94	0.68	0.83	0.77
3	0.82	1.94	1.39	1.10
4	0.86	1.57	1.25	1.03
5	0.81	2.07	1.44	1.18
6	0.84	1.73	1.32	1.11
7	0.91	1.01	1.00	0.93

As can be seen from Table 6, model complexity and prediction performance are not simply positively correlated. The Large Model (No. 2) exhibits the best performance, with *R*² reaching 0.94 and RMSE at 0.83°C, significantly outperforming the Benchmark Model (No. 1). In contrast, the Small Model (No. 3) performs the worst, with *R*² merely 0.82. This indicates that an appropriate increase in network capacity contributes to enhancing the model's ability to capture complex patterns in temperature series. Comparing Nos. 1, 4, and 2 reveals that when keeping the Dropout rate consistent, increasing the number of units in both the first and second layers enhances performance, with No. 2 (128−64) being the optimal configuration. Further comparison between Nos. 2 and 7 shows that increasing the number of units in the second layer from 32 to 64 yields a significant improvement. The impact of the Dropout rate can be observed by comparing Nos. 1 and 5: as the Dropout rate increases from 0.1 to 0.2, *R*² decreases from 0.84 to 0.81, indicating that an excessively high Dropout rate may lead to information loss, thereby impairing performance. Notably, the Dropout = 0.1 adopted by No. 2 has proven effective in preventing overfitting.

In summary, the optimal network structure is a two-layer LSTM with 128 units in the first layer, 64 units in the second layer, and a Dropout rate of 0.1. This configuration achieved the highest *R*² and the lowest RMSE on the test set, indicating its good generalization capability.

#### 4.2.2 Impact of time series window parameters on prediction performance.

Input window length and prediction step size are key factors influencing the effectiveness of time-series prediction. To investigate their impact on temperature prediction for the electrical cabinet, this study, based on the optimal network structure obtained in Section 4.2.1 ([128,64] with a Dropout rate of 0.1), designed seven representative combinations of input windows and prediction step sizes for comparative experiments, with specific configurations shown in Table 7. The test results of each combination under the same dataset are presented in Table 8.

**Table 7:** Configuration of sequence length parameters.

Combination No.	Input Window Length (minutes)	Prediction Step Size (minutes)
1	5	1
2	5	3
3	5	5
4	14	1
5	14	3
6	14	5
7	30	1

**Table 8:** Prediction results of different sequence length configurations (test set).

Combination No.	Input/Prediction	Input Window Length (minutes)	Prediction Step Size (minutes)	*R*²
1	Input: 5 min, Prediction: 1 min	5	1	0.96
2	Input: 5 min, Prediction: 3 min	5	3	0.92
3	Input: 5 min, Prediction: 3 min	5	5	0.86
4	Input: 14 min, Prediction: 1 min	14	1	0.94
5	Input: 14 min, Prediction: 3 min	14	3	0.92
6	Input: 14 min, Prediction: 5 min	14	5	0.89
7	Input: 30 min, Prediction: 1 min	30	1	0.89
**Combination No.**	**MSE**	**RMSE**	**MAE**	**Number of Samples (Training/Test)**
1	0.39	0.62	0.49	576/145
2	0.80	0.89	0.73	574/144
3	1.44	1.20	1.10	572/143
4	0.63	0.79	0.57	569/143
5	0.81	0.90	0.80	567/142
6	1.11	1.05	0.74	565/142
7	1.24	1.11	1.06	556/139

Based on the results of Table 8, the following conclusions can be drawn:

(1) Impact of Input Window Length: When the prediction step size is 1 minute, an input window length of 5 minutes performs best (*R*² = 0.96), outperforming 14 minutes (*R*² = 0.94) and 30 minutes (*R*² = 0.89). This indicates that the temperature series exhibits strong short-term correlations, and recent 5-minute historical data is sufficient to accurately predict the temperature of the next minute. Excessively long windows may introduce noise or redundant information, thereby degrading performance. For longer prediction step sizes (e.g., 5 minutes), an input window of 14 minutes performs relatively better (*R*² = 0.89), indicating that multi-step prediction requires longer historical information to capture trends.
(2) Impact of Prediction Step Size: As the prediction step size increases, the prediction accuracy of all configurations decreases significantly. For example, when the input window length is 5 minutes, as the prediction step size increases from 1 minute to 5 minutes, *R*² decreases from 0.96 to 0.86, and RMSE increases from 0.62 to 1.20°C. This pattern aligns with the fundamental characteristics of time-series prediction: the longer the prediction horizon, the greater the uncertainty.
(3) Optimal Combination: Comprehensive comparison indicates that the combination of an input window length of 5 minutes and a prediction step size of 1 minute (No. 1) exhibits the optimal performance in single-step prediction, with *R*² as high as 0.96 and RMSE only 0.62°C, meeting the accuracy and response speed requirements of real-time monitoring. For scenarios requiring multi-step prediction (e.g., early warning 5 minutes in advance), the combination of an input window length of 14 minutes and a prediction step size of 5 minutes (No. 6) is a preferable choice, with *R*² = 0.89 and RMSE = 1.05°C.

In summary, the optimal time window configuration for temperature prediction of the electrical cabinet in high-speed trains is to input 5-minute historical data to predict the temperature for the next 1 minute. This configuration fully leverages the short-term correlation of the temperature series, achieves the best balance between prediction accuracy and real-time performance, and is suitable for practical engineering applications. For long-term prediction needs, it is recommended to adopt an iterative prediction strategy to reduce error accumulation.

## 5. Conclusion

Addressing the engineering demands of accurate temperature prediction and early warning for key electrical components in the electrical cabinet of high-speed trains, this paper conducts an in-depth study on a temperature prediction model based on the Long Short-Term Memory (LSTM) network. Confronted with the challenge of long-term temporal dependencies embedded in temperature data under complex operating conditions, the study systematically accomplished tasks including temperature data acquisition, preprocessing, and comparative analysis of multiple models. Experimental results demonstrate that, compared with traditional and classical models such as the Radial Basis Function (RBF) neural network, ARIMA, and Prophet, the proposed LSTM model exhibits significant advantages in terms of prediction accuracy, stability, and generalization capability. The model can effectively capture the long-term development trends and short-term fluctuation characteristics of temperature changes in the electrical cabinet, and its excellent performance provides a solid technical foundation for accurately identifying potential temperature anomalies and achieving early fault warning. Meanwhile, through systematic parameter optimization, this paper determined the optimal LSTM model architecture and optimal time series window parameters, further enhancing prediction accuracy. Multi-scenario and multi-point validation confirmed the model's robustness across different operating conditions and measurement points. This study not only fills the research gap in the application of LSTM for temperature prediction of key electrical components in the electrical cabinet of high-speed trains, but also, through its constructed prediction framework and validation results, provides a reference scheme of significant practical application value for intelligent condition monitoring of high-speed train electrical equipment, formulation of proactive maintenance strategies, and ensuring the safety and reliability of train operation.

## Supporting information

S1 File Data. (XLS)
