# Supplementary material for: Research on temperature prediction model for key components in highspeed rail electrical cabinets based on LSTM
Source: PLoS One. 2026 Aug 17;21(8):e0355411. doi: 10.1371/journal.pone.0355411 (PMC13480594; doi:10.1371/journal.pone.0355411)
Supplement: Supplementary file 1 [file pone.0355411.s002.docx]

**Response to Reviewers**

**Manuscript ID:** PONE-D-26-17701

**Title:** Research on temperature prediction model for key components in highspeed rail electrical cabinets based on LSTM

**Journal:** PLOS ONE

Dear Dr. Zhang (Academic Editor) and Reviewers,

We sincerely thank you for your thorough and constructive comments on our manuscript. We have carefully considered all the points raised and have revised the manuscript accordingly. Below we provide a point-by-point response to each comment. All changes in the revised manuscript are highlighted in yellow (or marked with track changes in the separate file). We believe the revisions have substantially improved the technical soundness, experimental rigor, and clarity of the paper.

### Response to Academic Editor’s General Comments

**Comment:** Significant revisions are still needed regarding technical details, experimental verification, and writing coherence.

**Response:** We have addressed all concerns in detail. Specifically, we have (1) corrected the definition and formula of MAPE and added MAE and MaxRE; (2) resolved the data sample count discrepancy; (3) corrected the classification of the Prophet model; (4) enriched the literature review and added recommended references; (5) rephrased the research gap statement; (6) discussed the practical limitations of the predetermined speed curve; (7) supplemented experimental equipment details and environmental conditions; (8) provided a deeper analysis of model failures based on data characteristics; (9) added ablation study descriptions; (10) included multi-condition and multi-point validation; (11) clarified the LSTM gating mechanism in the context of temperature data; (12) detailed the error accumulation mitigation strategy; and (13) updated the funding and competing interests statements. All changes are clearly indicated in the revised manuscript.

### Response to Reviewer #1

**Comment 1:** The paper defines MAPE as the maximum absolute relative error whereas MAPE conventionally stands for mean absolute percentage error. The provided formula and interpretation are therefore incorrect and misleading.

**Response:** We apologize for this error. We have corrected the definition and formula. Now MAPE is defined as the Mean Absolute Percentage Error (Equation 6), and we introduced a separate metric MaxRE (Maximum Absolute Relative Error) to replace the original formula (Equation 7). The corresponding text has been revised in Section 2.3. All subsequent references to MAPE in the text and tables have been corrected accordingly.

**Comment 2:** The dataset contains 726 samples from approximately 12 hours of data collected at one sample per minute, which should yield only 720 samples. This numerical discrepancy is not explained and raises concerns about data consistency.

**Response:** We appreciate this observation. We have clarified in Section 3.1 that the recorder remained active for an additional 6 minutes before departure and after parking, capturing transient thermal responses during startup and shutdown phases. This yields 726 samples in total. The revised text now explicitly explains this deviation.

**Comment 3:** The Prophet model is described as a deep learning time-series prediction model, which is inaccurate. Prophet is a decomposable additive model with Bayesian inference and does not belong to the deep learning category.

**Response:** We agree and have corrected this classification. In Section 4.1, we now describe Prophet as "the decomposable time-series model Prophet with Bayesian inference" and removed the "deep learning" label.

**Comment 4:** LSTM is the key contribution. Its use in other field should be reviewed for highlighting usefulness. For instance, Remote condition monitoring of rail tracks using distributed acoustic sensing (DAS): A deep CNN-LSTM-SW based model; Feature selection-based irradiance forecast for efficient operation of a stand-alone PV system.

**Response:** We thank the reviewer for these valuable suggestions. We have added the recommended references (along with brief discussions) to the introduction, highlighting the successful applications of LSTM in related domains such as rail track monitoring and photovoltaic irradiance forecasting. This strengthens the motivation for adopting LSTM in our work. The added text is in the second paragraph of Section 1 (Introduction).

**Comment 5:** The introduction claims a research gap regarding LSTM application to electrical cabinet temperature prediction, yet several cited works already apply LSTM to similar electrical equipment. The claimed gap is thus overstated.

**Response:** We agree and have rephrased the claim. We now acknowledge that LSTM has been applied to transformers and substation equipment, but we emphasize that no prior study has specifically addressed the coupled temperature dynamics of key components (e.g., relays) inside high-speed rail electrical cabinets under varying operational conditions. This more precise statement better reflects the true novelty. The revision appears in the last paragraph of Section 1.

**Comment 6:** The rolling prediction mechanism relies on a predetermined future speed curve as input, which ignores real-world operational uncertainties such as speed deviations due to traffic or weather. The practical feasibility of this assumption is not adequately addressed.

**Response:** We acknowledge this limitation. In Section 2.1, we have added a discussion that within the short-term prediction window (e.g., 5 minutes), speed variations are typically limited and follow predictable patterns (e.g., approaching a station). For longer horizons or higher uncertainty scenarios, we note that the model could be extended with real-time speed predictions or robust interval forecasts, which we leave for future work. This clarifies the practical applicability of our assumption.

### Response to Reviewer #2

**Comment 1:** The literature review section of this study is insufficiently detailed. It merely lists the existing research results related to temperature prediction, without systematically summarizing the applicable scenarios, advantages, and limitations of each method.

**Response:** We have substantially rewritten the literature review in Section 1. Now we categorize existing methods into traditional statistical (ARIMA), machine learning (RBF), and deep learning (LSTM) approaches, and explicitly summarize their strengths, weaknesses, and suitable application scenarios. This provides a clearer context for our work.

**Comment 2:** The LSTM part of this paper has content omissions and inconsistent numbering. It does not provide a detailed explanation of the logic for addressing the error accumulation problem in the rolling prediction mechanism, and the analysis of the principle of combining the gating mechanism with temperature time series features is rather superficial.

**Response:** We have made the following improvements:

In Section 2.2, we added a paragraph explaining how the forget gate, input gate, and output gate physically correspond to retaining long-term warming trends, responding to recent speed changes, and generating predictions, respectively.

In Section 2.4 (Model Training), we detailed the scheduled sampling strategy used to mitigate error accumulation during multi-step rolling prediction. We also noted that the number of recursive steps is limited in practice to control error propagation, and empirical results show sublinear RMSE growth.

We have also corrected all inconsistent numbering throughout the manuscript.

**Comment 3:** The expression and definition of the evaluation indicators in this study are incorrect. The maximum absolute relative error is abbreviated as MAPE in the text, but MAPE actually refers to the mean absolute percentage error, and the concepts are confused. It is recommended to correct the indicator names and calculation formulas, and supplement common regression evaluation indicators such as MAE and average absolute percentage error.

**Response:** This is the same issue raised by Reviewer #1. We have corrected the definitions and formulas in Section 2.3. We now include RMSE, MAE, MAPE, and MaxRE as evaluation metrics. MAPE is now correctly defined as the mean absolute percentage error. We have also updated all result tables accordingly.

**Comment 4:** The data and experimental sample description in this study are not detailed enough. The measured data only explains the sampling duration and frequency, without detailing the experimental equipment and experimental conditions.

**Response:** We have expanded Section 3.1 with detailed information: the model and accuracy of the temperature recorder (±0.3°C, resolution 0.1°C), the type of patch probes (PT1000), the mounting method (high-temperature polyimide tape), the onboard ambient temperature sensor (±0.5°C), and the range of ambient temperatures during the experiment (18–26°C outside, 22–30°C inside the vehicle). This ensures full reproducibility.

**Comment 5:** The experimental design of this study lacks ablation experiments. When optimizing the LSTM structure and time window parameters, no complete ablation experiments were set up to verify the impact of a single parameter change one by one. The conclusion of parameter comparison is less persuasive.

**Response:** We have added a clear statement in Section 4.2 that the seven configurations in Table 5 are designed as an ablation study, varying one parameter at a time while keeping others fixed. We also provided a more detailed analysis of each parameter’s effect (e.g., comparing models No. 1, 2, 3 for layer size; No. 1 vs. No. 5 for Dropout; No. 2 vs. No. 7 for asymmetric layers). This strengthens the persuasiveness of our parameter selection.

**Comment 6:** This study only presents basic indicator results. It did not combine the non-stationary, strong non-linearity, and coupling influence of the temperature of the high-speed rail electrical cabinet data characteristics to deeply analyze the internal reasons for RBF overfitting, poor generalization ability of Prophet and ARIMA.

**Response:** We have added in-depth analyses for each comparison model in Section 4.1:

**RBF:** Overfitting occurs because it lacks temporal modeling and its localized kernels cannot generalize to unseen operating condition distributions.

**Prophet:** It incorrectly attributes temperature variations to calendar seasonality, whereas the actual dynamics are driven by real-time operational conditions (speed, mileage, ambient temperature).

**ARIMA:** Its linear autoregressive structure fails to capture nonlinear responses during acceleration and deceleration.
These explanations now explicitly link the model failures to the non-stationary and strongly coupled nature of the temperature data.

**Comment 7:** The condition division and scenario verification in this study are insufficient. The experiment only selected a single temperature measurement point for verification. It did not test the model performance for typical operating conditions such as train start-up, constant speed, acceleration and deceleration, and stop, as well as different environmental temperature scenarios. It cannot fully reflect the robustness of the model in complex conditions.

**Response:** We have added a new subsection (3.3) titled "Multi-scenario and multi-point validation". In this subsection, we evaluate the LSTM model on two additional measurement points (Points 6 and 9) and under three typical operating conditions: acceleration, constant-speed, and deceleration. A new table (Table 3) summarizes the RMSE, R2*R*2, and MAPE for each condition and point. The results confirm that the model maintains good performance across different conditions and components, demonstrating its robustness. We also note that the ambient temperature range was already provided in Section 3.1, and within that range, the model’s performance remained stable.

### Response to Journal Requirements

**Requirement 1 (Style):** We have revised the manuscript to meet PLOS ONE’s style requirements, including file naming and formatting. We have also ensured that Figure 1 is properly cited in the text (now in Section 2.1).

**Requirement 2 (Code sharing):** We have provided a statement in the Data Availability section indicating that the code will be made available via an anonymous GitHub repository upon acceptance, and we have included the relevant data as supplementary files.

**Requirement 3 (Data Availability Statement):** We have completed the Data Availability Statement in the submission form and also included it in the manuscript. All data are either in the supplementary file or described within the paper.

**Requirement 4 (Funding and Competing Interests):** We have amended both the Funding Statement and Competing Interests Statement. In the revised manuscript, we explicitly state that the authors are employed by CRRC TANGSHAN CO., LTD., that this commercial affiliation does not alter our adherence to PLOS ONE policies, and that the funder had no role in study design, data analysis, decision to publish, or manuscript preparation. The specific roles of authors are noted in the author contributions.

**Requirement 5 (Figure citation):** We have ensured that Figure 1 is now referenced in the text (Section 2.1).

**Requirement 6 (Reviewer-recommended citations):** We have evaluated the suggested references and incorporated them where relevant (see response to Reviewer #1, Comment 4).

We believe that the revised manuscript now meets the publication criteria of PLOS ONE. We are grateful for the reviewers’ valuable insights, which have significantly improved the quality of our work. We look forward to your favorable decision.

Yours sincerely,

Lihui Zhou (Corresponding Author)

On behalf of all co-authors

CRRC TANGSHAN CO., LTD., Tangshan, China

Email: zhoulihui0328@163.com

**Note:** In the separate file, we have also provided a marked-up copy (with track changes) and a clean copy of the revised manuscript, as requested.
