# Supplementary material for: Research on temperature prediction model for key components in highspeed rail electrical cabinets based on LSTM
Source: PLoS One. 2026 Aug 17;21(8):e0355411. doi: 10.1371/journal.pone.0355411 (PMC13480594; doi:10.1371/journal.pone.0355411)
Supplement: Peer review history

# Peer review: Decision Letter 0

**DOI:** 10.1371/journal.pone.0355411.r001

**Authors:** Lei Zhang

9 Jun 2026

Dear Dr. Zhou,

Please submit your revised manuscript by Jul 24 2026 11:59PM. If you will need more time than this to complete your revisions, please reply to this message or contact the journal office at plosone@plos.org . When you're ready to submit your revision, log on to https://www.editorialmanager.com/pone/ and select the 'Submissions Needing Revision' folder to locate your manuscript file.

- A letter that responds to each point raised by the academic editor and reviewer(s). You should upload this letter as a separate file labeled 'Response to Reviewers'.
- A marked-up copy of your manuscript that highlights changes made to the original version. You should upload this as a separate file labeled 'Revised Manuscript with Track Changes'.
- An unmarked version of your revised paper without tracked changes. You should upload this as a separate file labeled 'Manuscript'.

If you would like to make changes to your financial disclosure, please include your updated statement in your cover letter. Guidelines for resubmitting your figure files are available below the reviewer comments at the end of this letter.

If applicable, we recommend that you deposit your laboratory protocols in protocols.io to enhance the reproducibility of your results. Protocols.io assigns your protocol its own identifier (DOI) so that it can be cited independently in the future. For instructions see: https://journals.plos.org/plosone/s/submission-guidelines#loc-laboratory-protocols . Additionally, PLOS ONE offers an option for publishing peer-reviewed Lab Protocol articles, which describe protocols hosted on protocols.io. Read more information on sharing protocols at https://plos.org/protocols?utm_medium=editorial-email&utm_source=authorletters&utm_campaign=protocols .

As the corresponding author, your ORCID iD is verified in the submission system and will appear in the published article. PLOS supports the use of ORCID, and we encourage all coauthors to register for an ORCID iD and use it as well. Please encourage your coauthors to verify their ORCID iD within the submission system before final acceptance, as unverified ORCID iDs will not appear in the published article. *Only*   the individual author can complete the verification step; PLOS staff *cannot*   verify ORCID iDs on behalf of authors.

We look forward to receiving your revised manuscript.

Kind regards,

Lei Zhang, PhD

Academic Editor

PLOS One

Journal Requirements:

When submitting your revision, we need you to address these additional requirements.

1.Please ensure that your manuscript meets PLOS ONE's style requirements, including those for file naming. The PLOS ONE style templates can be found at

https://journals.plos.org/plosone/s/file?id=wjVg/PLOSOne_formatting_sample_main_body.pdf and

https://journals.plos.org/plosone/s/file?id=ba62/PLOSOne_formatting_sample_title_authors_affiliations.pdf

2. Please note that PLOS One has specific guidelines on code sharing for submissions in which author-generated code underpins the findings in the manuscript. In these cases, we expect all author-generated code to be made available without restrictions upon publication of the work. Please review our guidelines at https://journals.plos.org/plosone/s/materials-and-software-sharing#loc-sharing-code and ensure that your code is shared in a way that follows best practice and facilitates reproducibility and reuse.

3. Please provide a complete Data Availability Statement in the submission form, ensuring you include all necessary access information or a reason for why you are unable to make your data freely accessible. If your research concerns only data provided within your submission, please write "All data are in the manuscript and/or supporting information files" as your Data Availability Statement.

4. Thank you for stating the following in the Competing Interests section:

“The authors have declared that no competing interests exist.”

We note that one or more of the authors are employed by a commercial company: CRRC TANGSHAN CO., LTD., Tangshan, China

1.        Please provide an amended Funding Statement declaring this commercial affiliation, as well as a statement regarding the Role of Funders in your study. If the funding organization did not play a role in the study design, data collection and analysis, decision to publish, or preparation of the manuscript and only provided financial support in the form of authors' salaries and/or research materials, please review your statements relating to the author contributions, and ensure you have specifically and accurately indicated the role(s) that these authors had in your study. You can update author roles in the Author Contributions section of the online submission form.

Please also include the following statement within your amended Funding Statement.

“The funder provided support in the form of salaries for authors [insert relevant initials], but did not have any additional role in the study design, data collection and analysis, decision to publish, or preparation of the manuscript. The specific roles of these authors are articulated in the ‘author contributions’ section.”

If your commercial affiliation did play a role in your study, please state and explain this role within your updated Funding Statement.

2. Please also provide an updated Competing Interests Statement declaring this commercial affiliation along with any other relevant declarations relating to employment, consultancy, patents, products in development, or marketed products, etc.

Within your Competing Interests Statement, please confirm that this commercial affiliation does not alter your adherence to all PLOS ONE policies on sharing data and materials by including the following statement: "This does not alter our adherence to  PLOS ONE policies on sharing data and materials.” (as detailed online in our guide for authors http://journals.plos.org/plosone/s/competing-interests) . If this adherence statement is not accurate and  there are restrictions on sharing of data and/or materials, please state these. Please note that we cannot proceed with consideration of your article until this information has been declared.

Please include both an updated Funding Statement and Competing Interests Statement in your cover letter. We will change the online submission form on your behalf.

5. Please ensure that you refer to Figure 1 in your text as, if accepted, production will need this reference to link the reader to the figure.

6. If the reviewer comments include a recommendation to cite specific previously published works, please review and evaluate these publications to determine whether they are relevant and should be cited. There is no requirement to cite these works unless the editor has indicated otherwise.

Additional Editor Comments:

Significant revisions are still needed regarding technical details, experimental verification, and writing coherence.

[Note: HTML markup is below. Please do not edit.]

Reviewers' comments:

Reviewer's Responses to Questions

**Comments to the Author**

1. Is the manuscript technically sound, and do the data support the conclusions?

Reviewer #1: Partly

Reviewer #2: Yes

**********

2. Has the statistical analysis been performed appropriately and rigorously?

Reviewer #1: Yes

Reviewer #2: Yes

**********

3. Have the authors made all data underlying the findings in their manuscript fully available?

The PLOS Data policy

Reviewer #1: Yes

Reviewer #2: No

**********

4. Is the manuscript presented in an intelligible fashion and written in standard English?

Reviewer #1: Yes

Reviewer #2: Yes

**********

Reviewer #1: This study presents a temperature prediction model for key components in highspeed rail electrical cabinets based on LSTM. However, several critical issues need to be addressed before publication.

1. The paper defines MAPE as the maximum absolute relative error whereas MAPE conventionally stands for mean absolute percentage error. The provided formula and interpretation are therefore incorrect and misleading.

2. The dataset contains 726 samples from approximately 12 hours of data collected at one sample per minute, which should yield only 720 samples. This numerical discrepancy is not explained and raises concerns about data consistency.

3. The Prophet model is described as a deep learning time‑series prediction model, which is inaccurate. Prophet is a decomposable additive model with Bayesian inference and does not belong to the deep learning category.

4. LSTM is the key contribution. Its use in other field should be reviewed for highlighting usefulness. For instance, Remote condition monitoring of rail tracks using distributed acoustic sensing (DAS): A deep CNN-LSTM-SW based model; Feature selection-based irradiance forecast for efficient operation of a stand-alone PV system.

5. The introduction claims a research gap regarding LSTM application to electrical cabinet temperature prediction, yet several cited works already apply LSTM to similar electrical equipment. The claimed gap is thus overstated.

6. The rolling prediction mechanism relies on a predetermined future speed curve as input, which ignores real‑world operational uncertainties such as speed deviations due to traffic or weather. The practical feasibility of this assumption is not adequately addressed.

Reviewer #2: 1. The literature review section of this study is insufficiently detailed. It merely lists the existing research results related to temperature prediction, without systematically summarizing the applicable scenarios and inherent flaws of each model (RBF, ARIMA, Prophet, LSTM), nor clearly defining the innovation points and differentiation advantages of this study compared to similar LSTM temperature prediction literature.

2. The LSTM part of this paper has content omissions and inconsistent numbering. It does not provide a detailed explanation of the logic for addressing the error accumulation problem in the rolling prediction mechanism, and the analysis of the principle of combining the gating mechanism with temperature time series features is rather superficial.

3. The expression and definition of the evaluation indicators in this study are incorrect. The maximum absolute relative error is abbreviated as MAPE in the text, but MAPE actually refers to the mean absolute percentage error, and the concepts are confused. It is recommended to correct the indicator names and calculation formulas, and supplement common regression evaluation indicators such as MAE and average absolute percentage error.

4. The data and experimental sample description in this study are not detailed enough. The measured data only explains the sampling duration and frequency, without detailing the experimental equipment and experimental conditions.

5. The experimental design of this study lacks ablation experiments. When optimizing the LSTM structure and time window parameters, no complete ablation experiments were set up to verify the impact of a single parameter change one by one. The conclusion of parameter comparison is less persuasive.

6. This study only presents basic indicator results. It did not combine the non-stationary, strong non-linearity, and coupling influence of the temperature of the high-speed rail electrical cabinet data characteristics to deeply analyze the internal reasons for RBF overfitting, poor generalization ability of Prophet and ARIMA.

7. The condition division and scenario verification in this study are insufficient. The experiment only selected a single temperature measurement point for verification. It did not test the model performance for typical operating conditions such as train start-up, constant speed, acceleration and deceleration, and stop, as well as different environmental temperature scenarios. It cannot fully reflect the robustness of the model in complex conditions.

**********

what does this mean? ). If published, this will include your full peer review and any attached files.

If you choose “no”, your identity will remain anonymous but your review may still be made public.

**Do you want your identity to be public for this peer review?**   For information about this choice, including consent withdrawal, please see our Privacy Policy

Reviewer #1: No

Reviewer #2: No

**********

[NOTE: If reviewer comments were submitted as an attachment file, they will be attached to this email and accessible via the submission site. Please log into your account, locate the manuscript record, and check for the action link "View Attachments". If this link does not appear, there are no attachment files.]

To ensure your figures meet our technical requirements, please review our figure guidelines: https://journals.plos.org/plosone/s/figures

You may also use PLOS’s free figure tool, NAAS, to help you prepare publication quality figures: https://journals.plos.org/plosone/s/figures#loc-tools-for-figure-preparation.

NAAS will assess whether your figures meet our technical requirements by comparing each figure against our figure specifications.

# Author comment: Author response to Decision Letter 1

**DOI:** 10.1371/journal.pone.0355411.r002

27 Jun 2026

Response to Reviewers

Manuscript ID: PONE-D-26-17701

Title: Research on temperature prediction model for key components in highspeed rail electrical cabinets based on LSTM

Journal: PLOS ONE

Dear Dr. Zhang (Academic Editor) and Reviewers,

We sincerely thank you for your thorough and constructive comments on our manuscript. We have carefully considered all the points raised and have revised the manuscript accordingly. Below we provide a point-by-point response to each comment. All changes in the revised manuscript are highlighted in yellow (or marked with track changes in the separate file). We believe the revisions have substantially improved the technical soundness, experimental rigor, and clarity of the paper.

Response to Academic Editor’s General Comments

Comment: Significant revisions are still needed regarding technical details, experimental verification, and writing coherence.

Response: We have addressed all concerns in detail. Specifically, we have (1) corrected the definition and formula of MAPE and added MAE and MaxRE; (2) resolved the data sample count discrepancy; (3) corrected the classification of the Prophet model; (4) enriched the literature review and added recommended references; (5) rephrased the research gap statement; (6) discussed the practical limitations of the predetermined speed curve; (7) supplemented experimental equipment details and environmental conditions; (8) provided a deeper analysis of model failures based on data characteristics; (9) added ablation study descriptions; (10) included multi-condition and multi-point validation; (11) clarified the LSTM gating mechanism in the context of temperature data; (12) detailed the error accumulation mitigation strategy; and (13) updated the funding and competing interests statements. All changes are clearly indicated in the revised manuscript.

Response to Reviewer #1

Comment 1: The paper defines MAPE as the maximum absolute relative error whereas MAPE conventionally stands for mean absolute percentage error. The provided formula and interpretation are therefore incorrect and misleading.

Response: We apologize for this error. We have corrected the definition and formula. Now MAPE is defined as the Mean Absolute Percentage Error (Equation 6), and we introduced a separate metric MaxRE (Maximum Absolute Relative Error) to replace the original formula (Equation 7). The corresponding text has been revised in Section 2.3. All subsequent references to MAPE in the text and tables have been corrected accordingly.

Comment 2: The dataset contains 726 samples from approximately 12 hours of data collected at one sample per minute, which should yield only 720 samples. This numerical discrepancy is not explained and raises concerns about data consistency.

Response: We appreciate this observation. We have clarified in Section 3.1 that the recorder remained active for an additional 6 minutes before departure and after parking, capturing transient thermal responses during startup and shutdown phases. This yields 726 samples in total. The revised text now explicitly explains this deviation.

Comment 3: The Prophet model is described as a deep learning time-series prediction model, which is inaccurate. Prophet is a decomposable additive model with Bayesian inference and does not belong to the deep learning category.

Response: We agree and have corrected this classification. In Section 4.1, we now describe Prophet as "the decomposable time-series model Prophet with Bayesian inference" and removed the "deep learning" label.

Comment 4: LSTM is the key contribution. Its use in other field should be reviewed for highlighting usefulness. For instance, Remote condition monitoring of rail tracks using distributed acoustic sensing (DAS): A deep CNN-LSTM-SW based model; Feature selection-based irradiance forecast for efficient operation of a stand-alone PV system.

Response: We thank the reviewer for these valuable suggestions. We have added the recommended references (along with brief discussions) to the introduction, highlighting the successful applications of LSTM in related domains such as rail track monitoring and photovoltaic irradiance forecasting. This strengthens the motivation for adopting LSTM in our work. The added text is in the second paragraph of Section 1 (Introduction).

Comment 5: The introduction claims a research gap regarding LSTM application to electrical cabinet temperature prediction, yet several cited works already apply LSTM to similar electrical equipment. The claimed gap is thus overstated.

Response: We agree and have rephrased the claim. We now acknowledge that LSTM has been applied to transformers and substation equipment, but we emphasize that no prior study has specifically addressed the coupled temperature dynamics of key components (e.g., relays) inside high-speed rail electrical cabinets under varying operational conditions. This more precise statement better reflects the true novelty. The revision appears in the last paragraph of Section 1.

Comment 6: The rolling prediction mechanism relies on a predetermined future speed curve as input, which ignores real-world operational uncertainties such as speed deviations due to traffic or weather. The practical feasibility of this assumption is not adequately addressed.

Response: We acknowledge this limitation. In Section 2.1, we have added a discussion that within the short-term prediction window (e.g., 5 minutes), speed variations are typically limited and follow predictable patterns (e.g., approaching a station). For longer horizons or higher uncertainty scenarios, we note that the model could be extended with real-time speed predictions or robust interval forecasts, which we leave for future work. This clarifies the practical applicability of our assumption.

Response to Reviewer #2

Comment 1: The literature review section of this study is insufficiently detailed. It merely lists the existing research results related to temperature prediction, without systematically summarizing the applicable scenarios, advantages, and limitations of each method.

Response: We have substantially rewritten the literature review in Section 1. Now we categorize existing methods into traditional statistical (ARIMA), machine learning (RBF), and deep learning (LSTM) approaches, and explicitly summarize their strengths, weaknesses, and suitable application scenarios. This provides a clearer context for our work.

Comment 2: The LSTM part of this paper has content omissions and inconsistent numbering. It does not provide a detailed explanation of the logic for addressing the error accumulation problem in the rolling prediction mechanism, and the analysis of the principle of combining the gating mechanism with temperature time series features is rather superficial.

Response: We have made the following improvements:

In Section 2.2, we added a paragraph explaining how the forget gate, input gate, and output gate physically correspond to retaining long-term warming trends, responding to recent speed changes, and generating predictions, respectively.

In Section 2.4 (Model Training), we detailed the scheduled sampling strategy used to mitigate error accumulation during multi-step rolling prediction. We also noted that the number of recursive steps is limited in practice to control error propagation, and empirical results show sublinear RMSE growth.

We have also corrected all inconsistent numbering throughout the manuscript.

Comment 3: The expression and definition of the evaluation indicators in this study are incorrect. The maximum absolute relative error is abbreviated as MAPE in the text, but MAPE actually refers to the mean absolute percentage error, and the concepts are confused. It is recommended to correct the indicator names and calculation formulas, and supplement common regression evaluation indicators such as MAE and average absolute percentage error.

Response: This is the same issue raised by Reviewer #1. We have corrected the definitions and formulas in Section 2.3. We now include RMSE, MAE, MAPE, and MaxRE as evaluation metrics. MAPE is now correctly defined as the mean absolute percentage error. We have also updated all result tables accordingly.

Comment 4: The data and experimental sample description in this study are not detailed enough. The measured data only explains the sampling duration and frequency, without detailing the experimental equipment and experimental conditions.

Response: We have expanded Section 3.1 with detailed information: the model and accuracy of the temperature recorder (±0.3°C, resolution 0.1°C), the type of patch probes (PT1000), the mounting method (high-temperature polyimide tape), the onboard ambient temperature sensor (±0.5°C), and the range of ambient temperatures during the experiment (18–26°C outside, 22–30°C inside the vehicle). This ensures full reproducibility.

Comment 5: The experimental design of this study lacks ablation experiments. When optimizing the LSTM structure and time window parameters, no complete ablation experiments were set up to verify the impact of a single parameter change one by one. The conclusion of parameter comparison is less persuasive.

Response: We have added a clear statement in Section 4.2 that the seven configurations in Table 5 are designed as an ablation study, varying one parameter at a time while keeping others fixed. We also provided a more detailed analysis of each parameter’s effect (e.g., comparing models No. 1, 2, 3 for layer size; No. 1 vs. No. 5 for Dropout; No. 2 vs. No. 7 for asymmetric layers). This strengthens the persuasiveness of our parameter selection.

Comment 6: This study only presents basic indicator results. It did not combine the non-stationary, strong non-linearity, and coupling influence of the temperature of the high-speed rail electrical cabinet data characteristics to deeply analyze the internal reasons for RBF overfitting, poor generalization ability of Prophet and ARIMA.

Response: We have added in-depth analyses for each comparison model in Section 4.1:

RBF: Overfitting occurs because it lacks temporal modeling and its localized kernels cannot generalize to unseen operating condition distributions.

Prophet: It incorrectly attributes temperature variations to calendar seasonality, whereas the actual dynamics are driven by real-time operational conditions (speed, mileage, ambient temperature).

ARIMA: Its linear autoregressive structure fails to capture nonlinear responses during acceleration and deceleration.

These explanations now explicitly link the model failures to the non-stationary and strongly coupled nature of the temperature data.

Comment 7: The condition division and scenario verification in this study are insufficient. The experiment only selected a single temperature measurement point for verification. It did not test the model performance for typical operating conditions such as train start-up, constant speed, acceleration and deceleration, and stop, as well as different environmental temperature scenarios. It cannot fully reflect the robustness of the model in complex conditions.

Response: We have added a new subsection (3.3) titled "Multi-scenario and multi-point validation". In this subsection, we evaluate the LSTM model on two additional measurement points (Points 6 and 9) and under three typical operating conditions: acceleration, constant-speed, and deceleration. A new table (Table 3) summarizes the RMSE, R2R2, and MAPE for each condition and point. The results confirm that the model maintains good performance across different conditions and components, demonstrating its robustness. We also note that the ambient temperature range was already provided in Section 3.1, and within that range, the model’s performance remained stable.

Response to Journal Requirements

Requirement 1 (Style): We have revised the manuscript to meet PLOS ONE’s style requirements, including file naming and formatting. We have also ensured that Figure 1 is properly cited in the text (now in Section 2.1).

Requirement 2 (Code sharing): We have provided a statement in the Data Availability section indicating that the code will be made available via an anonymous GitHub repository upon acceptance, and we have included the relevant data as supplementary files.

Requirement 3 (Data Availability Statement): We have completed the Data Availability Statement in the submission form and also included it in the manuscript. All data are either in the supplementary file or described within the paper.

Requirement 4 (Funding and Competing Interests): We have amended both the Funding Statement and Competing Interests Statement. In the revised manuscript, we explicitly state that the authors are employed by CRRC TANGSHAN CO., LTD., that this commercial affiliation does not alter our adherence to PLOS ONE policies, and that the funder had no role in study design, data analysis, decision to publish, or manuscript preparation. The specific roles of authors are noted in the author contributions.

Requirement 5 (Figure citation): We have ensured that Figure 1 is now referenced in the text (Section 2.1).

Requirement 6 (Reviewer-recommended citations): We have evaluated the suggested references and incorporated them where relevant (see response to Reviewer #1, Comment 4).

We believe that the revised manuscript now meets the publication criteria of PLOS ONE. We are grateful for the reviewers’ valuable insights, which have significantly improved the quality of our work. We look forward to your favorable decision.

Yours sincerely,

Lihui Zhou (Corresponding Author)

On behalf of all co-authors

CRRC TANGSHAN CO., LTD., Tangshan, China

Email: zhoulihui0328@163.com

Note: In the separate file, we have also provided a marked-up copy (with track changes) and a clean copy of the revised manuscript, as requested.

Attachment Submitted filename: Response to Reviewers.docx

# Peer review: Decision Letter 1

**DOI:** 10.1371/journal.pone.0355411.r003

**Authors:** Lei Zhang

21 Jul 2026

Research on temperature prediction model for key components in highspeed rail electrical cabinets based on LSTM

PONE-D-26-17701R1

Dear Dr. Zhou,

We’re pleased to inform you that your manuscript has been judged scientifically suitable for publication and will be formally accepted for publication once it meets all outstanding technical requirements.

Within one week, you’ll receive an e-mail detailing the required amendments. When these have been addressed, you’ll receive a formal acceptance letter and your manuscript will be scheduled for publication.

An invoice will be generated when your article is formally accepted. Please note, if your institution has a publishing partnership with PLOS and your article meets the relevant criteria, all or part of your publication costs will be covered. Please make sure your user information is up-to-date by logging into Editorial Manager at Editorial Manager®  and clicking the ‘Update My Information' link at the top of the page. For questions related to billing, please contact billing support .

If your institution or institutions have a press office, please notify them about your upcoming paper to help maximize its impact. If they’ll be preparing press materials, please inform our press team as soon as possible -- no later than 48 hours after receiving the formal acceptance. Your manuscript will remain under strict press embargo until 2 pm Eastern Time on the date of publication. For more information, please contact onepress@plos.org.

Kind regards,

Lei Zhang, PhD

Academic Editor

PLOS One

Additional Editor Comments (optional):

The revised paper is publishable.

Reviewers' comments:

Reviewer's Responses to Questions

**Comments to the Author**

Reviewer #1: (No Response)

Reviewer #2: (No Response)

**********

2. Is the manuscript technically sound, and do the data support the conclusions?

Reviewer #1: (No Response)

Reviewer #2: (No Response)

**********

3. Has the statistical analysis been performed appropriately and rigorously?

Reviewer #1: (No Response)

Reviewer #2: (No Response)

**********

4. Have the authors made all data underlying the findings in their manuscript fully available?

The PLOS Data policy

Reviewer #1: (No Response)

Reviewer #2: (No Response)

**********

5. Is the manuscript presented in an intelligible fashion and written in standard English?

Reviewer #1: (No Response)

Reviewer #2: (No Response)

**********

Reviewer #1: (No Response)

Reviewer #2: (No Response)

**********

what does this mean? ). If published, this will include your full peer review and any attached files.

If you choose “no”, your identity will remain anonymous but your review may still be made public.

**Do you want your identity to be public for this peer review?**   For information about this choice, including consent withdrawal, please see our Privacy Policy

Reviewer #1: No

Reviewer #2: No

**********

# Editor report: Acceptance letter

**DOI:** 10.1371/journal.pone.0355411.r004

**Authors:** Lei Zhang

PONE-D-26-17701R1

PLOS One

Dear Dr. Zhou,

I'm pleased to inform you that your manuscript has been deemed suitable for publication in PLOS One. Congratulations! Your manuscript is now being handed over to our production team.

At this stage, our production department will prepare your paper for publication. This includes ensuring the following:

* All references, tables, and figures are properly cited

* All relevant supporting information is included in the manuscript submission,

* There are no issues that prevent the paper from being properly typeset

You will receive further instructions from the production team, including instructions on how to review your proof when it is ready. Please keep in mind that we are working through a large volume of accepted articles, so please give us a few days to review your paper and let you know the next and final steps.

Lastly, if your institution or institutions have a press office, please let them know about your upcoming paper now to help maximize its impact. If they'll be preparing press materials, please inform our press team within the next 48 hours. Your manuscript will remain under strict press embargo until 2 pm Eastern Time on the date of publication. For more information, please contact onepress@plos.org.

You will receive an invoice from PLOS for your publication fee after your manuscript has reached the completed accept phase. If you receive an email requesting payment before acceptance or for any other service, this may be a phishing scheme. Learn how to identify phishing emails and protect your accounts at https://explore.plos.org/phishing.

If we can help with anything else, please email us at customercare@plos.org.

Thank you for submitting your work to PLOS ONE and supporting open access.

Kind regards,

PLOS ONE Editorial Office Staff

on behalf of

Dr. Lei Zhang

Academic Editor

PLOS One
